# Plasma Cell–Free DNA Next-Generation Sequencing to Diagnose and Monitor Infections in Allogeneic Hematopoietic Stem Cell Transplant Patients

**DOI:** 10.1093/ofid/ofy301

**Published:** 2018-11-16

**Authors:** Monica Fung, Simona Zompi, Hon Seng, Desiree Hollemon, Adama Parham, David K Hong, Sivan Bercovici, Estelle Dolan, Kathy Lien, Justin Teraoka, Aaron C Logan, Peter Chin-Hong

**Affiliations:** 1 Division of Infectious Diseases, University of California San Francisco, San Francisco, California; 2 Karius, Inc., Redwood City, California; 3 School of Medicine, University of California Davis, Sacramento, California; 4 Division of Hematology and Blood and Marrow Transplantation, University of California San Francisco, San Francisco, California

**Keywords:** immunocompromised, molecular diagnostics, next-generation sequencing, stem cell transplant

## Abstract

Allogeneic hematopoietic stem cell transplant patients are at risk for common and atypical infections. Superior diagnostics can decrease infection-related morbidity and mortality. A novel plasma cell–free DNA next-generation sequencing test detected an uncommon presentation of *Chlamydia trachomatis* and recurrent and metastatic complications of *Staphylococcus aureus* bacteremia before standard microbiology.

Allogeneic hematopoietic stem cell transplantation (allo-HSCT) is a life-saving treatment for patients with hematologic malignancy. However, it increases the risk of infections, which cause significant morbidity and mortality in this population [1]. Diagnosing infections in allo-HSCT patients is challenging due to uncommon causative pathogens and atypical clinical presentations of disease.

Next-generation sequencing (NGS) holds promise as an infectious disease diagnostic that can quickly and accurately detect a wide range of pathogens to help guide therapy and monitor response [2]. Karius, Inc., has developed a novel plasma NGS platform that provides rapid and unbiased identification of >1000 pathogens [3]. We present a series of allo-HSCT cases where plasma NGS detected pathogens or complications of infection before standard microbiology.

## METHODS

Adult patients undergoing allo-HSCT for hematologic malignancy at the University of California, San Francisco (UCSF) were consented and enrolled in the Diagnosis of Infection in Stem Cell Transplant Patients OVER Time (DISCOVER) trial (NCT02804464). The protocol was approved by the UCSF Institutional Review Board (IRB#1518026). Patients were free of known active infections at initiation of their transplant conditioning. Patients underwent plasma sampling before conditioning, at the time of graft infusion, and at standard weekly intervals post-transplant, with additional sampling during acute illness.

**Figure 1.**
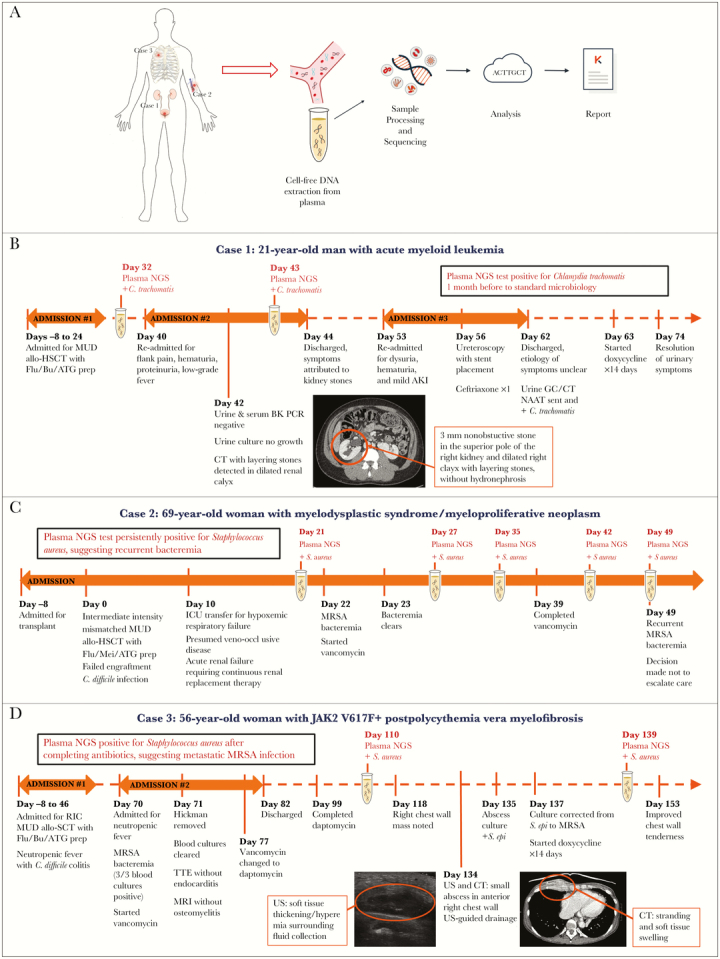
A, Workflow of Karius diagnostic test for infectious disease using NGS of plasma cell-free DNA. B, Timeline of events for Case 1. C, Timeline of events for Case 2. D, Timeline of events for Case 3. For the plasma NGS test, the day of plasma collection is shown. Abbreviations: AKI, acute kidney injury; allo-HSCT, allogeneic hematopoietic stem cell transplant; ATG, antithymocyte globulin; BK PCR, BK virus polymerase chain reaction test; Bu, busulfan; CT, computed tomography; Flu, fludarabine; GC/CT NAAT, gonorrhea and chlamydia nucleic acid amplification test; ICU, intensive care unit; Mel, melphalan; MRI, magnetic resonance imaging; MRSA, methicillin-resistant *Staphylococcus aureus*; MUD, matched unrelated donor; NGS, next-generation sequencing; PCR, polymerase chain reaction; RIC, reduced intensity conditioning; TTE, transthoracic echocardiogram; US, ultrasound.

Samples were transferred to the Karius CLIA/CAP Laboratory (Redwood City, CA) for processing. Cell-free DNA was extracted from plasma, NGS libraries were prepared, and sequencing was performed on an Illumina NextSeq 500 (Figure 1A). Sequencing reads identified as human were removed, and the remaining sequences were aligned to a curated pathogen database. Validated organisms found to be present above a predefined statistical threshold were reported as previously described [3–5]. The process from sample receipt to result typically takes 28 hours.

Cases were selected from the first 20 subjects (70 total) enrolled in the DISCOVER trial. Research study results were not made available to the clinical providers in real time and were not applied in clinical decision-making. For details, see the Supplementary Data.

## CASE REPORTS

### Case 1

A 21-year-old man with acute myelogenous leukemia and horseshoe kidney underwent human leukocyte antigen–matched unrelated donor myeloablative allo-HSCT. He was admitted on post-transplant days 40–44 for flank pain, hematuria, and low-grade fever (Figure 1B). History was negative for sexual activity in the prior 6 months. Work-up was notable for urinalysis showing hemoglobin and leukocyte esterase, urine culture without growth, and negative urine and serum BK virus polymerase chain reaction (PCR). Computed tomography (CT) of the abdomen and pelvis demonstrated a 3-mm nonobstructive stone in the superior pole of the right kidney and dilated right calyx with layering stones without hydronephrosis. Urology considered nephrolithiasis the most likely explanation for the patient’s symptoms, and he was discharged on alpha-1 antagonist treatment.

The patient was readmitted on post-transplant days 53–62 for ongoing dysuria and hematuria (Figure 1B). Labwork was notable for mild acute kidney injury; urinalysis showing hemoglobin, protein, and leukocyte esterase; urine culture without growth; and negative urine and serum BK virus PCRs. Renal ultrasound revealed a dilated right calyx with small stone fragments without hydronephrosis. Ureteroscopy was performed and revealed diffuse bladder inflammation with mild to moderate dilation of the intrarenal collecting system. Placement of a right-sided ureteral stent did not improve the patient’s symptoms, so it was removed 48 hours later. On discharge, the etiology of the patient’s symptoms remained unclear, so a urine gonorrhea/chlamydia nucleic acid amplification test was sent, returning positive for *Chlamydia trachomatis*. At outpatient follow-up 2 days postdischarge, the patient was started on a 14-day course of doxycycline with resolution of his urinary symptoms.

Plasma NGS was positive for *Chlamydia trachomatis* 7 days before initial symptoms and 30 days before standard microbiologic diagnosis (post-transplant day 32), as well as a day before discharge from the first admission (post-transplant day 43).

### Case 2

A 69-year-old woman with myelodysplastic syndrome/myeloproliferative neoplasm underwent intermediate-intensity mismatched unrelated donor allo-HSCT. Her post-transplant course was complicated by engraftment failure, *Clostridium difficile* infection, veno-occlusive disease, renal failure, and respiratory failure, requiring a prolonged course of intensive care (Figure 1C).

On post-transplant day 22, the patient developed methicillin-resistant *Staphylococcus aureus* (MRSA) bacteremia. After starting intravenous (IV) vancomycin, blood cultures cleared within 1 day (post-transplant day 23), and she received a 2-week course that ended on post-transplant day 39. On post-transplant day 49, blood cultures drawn for worsening hemodynamic instability showed recurrent MRSA bacteremia. Given poor oncologic prognosis, the patient was transitioned to comfort-focused care and died.

Plasma NGS testing was positive for *S. aureus* the day before initial MRSA bacteremia (post-transplant day 21), throughout the vancomycin course (post-transplant days 27 and 35), after stopping vancomycin (post-transplant day 42), and on the same day as recurrent MRSA bacteremia.

### Case 3

A 56-year-old woman with JAK2 V617F+ postpolycythemia vera myelofibrosis underwent reduced-intensity conditioning unrelated donor allo-HSCT, with her immediate post-transplant course complicated by neutropenic fever and *C. difficile* colitis (Figure 1D). On post-transplant days 70–82, the patient was readmitted for neutropenic fever and found to have MRSA bacteremia. After starting IV vancomycin, blood cultures cleared within 48 hours. The patient’s Hickman catheter was removed, and transthoracic echocardiogram and magnetic resonance imaging of the spine showed no endocarditis or osteomyelitis/discitis. Due to subtherapeutic serum levels, vancomycin was changed to daptomycin, and the patient completed a 4-week course of antibiotic therapy (through post-transplant day 99).

On post-transplant day 118, the patient presented to clinic reporting a right chest wall mass. On post-transplant day 134, ultrasound showed soft tissue thickening/hyperemia surrounding a 2.6 × 0.7 × 2.7-cm fluid collection, and chest CT demonstrated stranding and soft tissue swelling of the right anterior chest wall. Bacterial culture from same-day ultrasound-guided aspiration preliminarily identified *Staphylococcus epidermidis*. On post-transplant day 137, speciation was corrected to MRSA, and the patient was started on a 14-day course of oral doxycycline with improvement in chest wall tenderness.

Plasma NGS remained positive for *S. aureus* after completion of daptomycin for bacteremia (post-transplant day 110) and preceding the clinical and microbiological diagnosis of chest wall abscess (post-transplant day 139), becoming negative after drainage and antibiotics for abscess.

## DISCUSSION

Among allo-HSCT recipients, infections are often severe but lack classic symptoms such as fever and leukocytosis [1]. Current laboratory methods are frequently unable to establish a microbiologic diagnosis [1, 6]. We describe 3 cases where plasma NGS could have facilitated early identification of an uncommon presentation of *Chlamydia trachomatis* and indicated persistent MRSA infection before microbiologic diagnosis of recurrent bacteremia and metastatic infection.

In Case 1, given the patient’s atypical presentation, directed chlamydia testing was not sent until the end of his second admission. Although urethritis with dysuria and leukocyturia is the most common presentation of chlamydia infection in men, hematuria and proteinuria have been described in pediatric and adolescent males with chlamydia urethritis [7, 8]. Plasma NGS testing was positive 19 and 30 days before standard microbiology and could have potentially prevented a 10-day hospitalization with invasive ureteroscopy with stent placement.

In Case 2, plasma NGS remained positive for *S. aureus* after completion of therapy for MRSA bacteremia and before identification of recurrent bacteremia by standard culture. The incidence of bacteremia among allo-HSCT patients is estimated to be as high as 39% [9]. *S. aureus* bacteremia, in particular, is associated with significant morbidity and mortality, with recurrence occurring in 2%–17% of cases, especially among patients with MRSA [10]. Among allo-HSCT recipients, risk factors for recurrent *S. aureus* bacteremia include graft-vs-host disease, corticosteroid treatment, liver dysfunction, and prolonged hospitalization [11]. As plasma NGS may detect pathogens 2–5 days before bacteremia and remains positive on average 3 days longer than blood culture, plasma NGS results could have provided an early indication of recurrent or relapsed bacteremia [12, 13].

In Case 3, plasma NGS remained positive after treatment for MRSA bacteremia and before clinical diagnosis of chest wall abscess. Metastatic infection is a common complication of *S. aureus* bacteremia, occurring in up to 39% of patients [14, 15]. This immunocompromised patient exhibited limited symptoms, and the causative organism was initially erroneously identified as *S. epidermidis,* contributing to delayed treatment. Persistent positive plasma NGS testing for *S. aureus* despite completion of antibiotics could have indicated persistent occult infection.

Beyond the detection of active infection, plasma NGS testing reports other relevant pathogens for allo-HSCT monitoring. In Case 1, routine plasma NGS was positive for cytomegalovirus (CMV) on post-transplant days 14–32, coinciding with standard CMV PCR. In Case 2, plasma NGS detected human herpes virus 6B, *Pneumocystis jirovecii,* and CMV on post-transplant day 42. Due to a decision not to escalate care, standard microbiology tests were not obtained. In Case 3, plasma NGS showed CMV on post-transplant days 35–64 and Epstein-Barr virus (EBV) on post-transplant day 42, corresponding to standard CMV and EBV PCRs, respectively.

In summary, plasma NGS is a rapid, noninvasive, and unbiased test that detects a broad range of pathogens from a single plasma sample [3–5]. Here, we describe cases where plasma NGS not only identified compartmentalized non-bloodstream infections, but also detected persistent pathogen DNA after bacteremia that could have served as early indications of recurrent or occult infection. In immunocompromised patients such as allo-HSCT recipients, plasma NGS may aid in the diagnosis of infections caused by uncommon pathogens and in situations of atypical clinical presentations, potentially allowing for early targeted therapy to improve clinical outcomes and decrease antimicrobial resistance and drug toxicity. Further study is needed to fully define the test characteristics of plasma NGS.

## Supplementary Data

Supplementary materials are available at *Open Forum Infectious Diseases* online. Consisting of data provided by the authors to benefit the reader, the posted materials are not copyedited and are the sole responsibility of the authors, so questions or comments should be addressed to the corresponding author.

ofy301_suppl_supplementary_appendix
