# Supplementary material for: Plasma Cell–Free DNA Next-Generation Sequencing to Diagnose and Monitor Infections in Allogeneic Hematopoietic Stem Cell Transplant Patients
Source: Open Forum Infect Dis. 2018 Nov 16;5(12):ofy301. doi: 10.1093/ofid/ofy301 (PMC6297859; doi:10.1093/ofid/ofy301)
Supplement: ofy301_suppl_supplementary_appendix [file ofy301_suppl_supplementary_appendix.docx]

**SUPPLEMENTAL APPENDIX**

**The Karius^®^ Test**

***Reference Database and QC***

Reference genomes for Homo sapiens and microorganisms (bacteria, viruses, fungi/molds, and other eukaryotic pathogens) were retrieved from the National Center for Biotechnology Information (NCBI) ftp site (NCBI, U.S. National Library of Medicine (NLM), Human Genome: <https://www.ncbi.nlm.nih.gov/genome/guide/human/>, release GRCh38.p7), ( NCBI, U.S. NLM, Microbial Genomes: <https://www.ncbi.nlm.nih.gov/genome/microbes/>). Sequence similarities between microorganism references were inspected to identify taxonomic mislabeling and sequence contamination. From the reference genomes passing these quality controls, a subset was selected to maximize sequence diversity. As part of the selection process, NCBI BioSample data (NCBI, U.S. NLM, Biosample: https://www.ncbi.nlm.nih.gov/biosample/), were used to ensure the inclusion of reference genomes from both clinical and non-clinical isolates. The final reference genome dataset included over 21,000 reference genomes, containing over 2.7 million sequences. Selected sequences were collected into a single FASTA file and used to generate our microorganism reference BLAST database. A subset of these taxa, including 1251 clinically significant microorganisms, was used as the clinical reportable range.

***Clinically Reportable Range (CRR)***

The selection of organisms in our clinically reportable range (CRR) was performed as follows. A candidate list was generated by two board-certified Infectious Disease physicians by including (a) DNA viruses, culturable bacteria, additional fastidious and unculturable bacteria, mycobacteria, and eukaryotic pathogens from the standard text [1] and a number of infectious disease references, (b) organisms referenced in published case reports and (c) reference genomes sequenced from human clinical isolates (as indicated by NCBI’s BioSample resource) with publications supporting pathogenicity. Organisms from the above list that were associated with high quality reference genomes, as determined by our reference database QC process (see above), were used to further narrow the range. Finally, organisms at risk of generating common false-positive calls because of sporadic environmental contamination were removed. The sequence database is continuously curated to minimize human cross-reactivity as well as cross-reactivity between pathogens and is screened to mitigate contamination with sequences from human or other organisms.

***Sequencing***

Plasma samples were thawed, centrifuged at 16,000 rcf for ten minutes, and spiked with a known concentration of synthetic DNA molecules for quality control purposes. Cell-free DNA was extracted from 0.5 mL plasma using a magnetic bead-based method (Omega Biotek, Norcross, GA). DNA libraries for sequencing are constructed using a modified Ovation® Ultralow System V2 library preparation kit (NuGEN, San Carlos, CA). Negative controls (buffer only instead of plasma) and positive controls (healthy plasma spiked with a known mixture of microbial DNA fragments) were processed alongside patient samples in every batch. Samples were multiplexed with other samples and sequenced on an Illumina NextSeq® 500. Sequencing depth was approximately 79 million reads per sample on average, with a range of 3 million to 322 million reads.

***Analysis Pipeline***

Primary sequencing output files were processed using bcl2fastq (v2.17.1.14) to generate the demultiplexed sequencing reads files. Reads were filtered based on sequencing quality and trimmed based on partial or full adapter sequence. The bowtie2 (version 2.2.4) tool was used to align the remaining reads against Karius' human and synthetic-molecules references. Sequencing reads exhibiting strong alignment against the human references or the synthetic molecule references were collected and excluded from further analysis. Remaining reads were aligned against Karius' proprietary microorganism reference database using NCBI-blast (version 2.2.30+). A mixture model was used to assign a likelihood to the complete collection of sequencing reads that included the read sequence probabilities and the (unknown) abundances of each taxon in the sample. An expectation-maximization algorithm was applied to compute the maximum likelihood estimate of each taxon abundance. Only taxa whose abundances rejected the null hypothesis of originating from environmental contamination

(as calculated from the negative controls) at high significance levels were reported. The quantity for each organism identified was expressed in Molecules Per Microliter (MPM), the number of DNA sequencing reads from the reported organism present per microliter of plasma. The entire process from DNA extraction through analysis was typically completed within 28 hours.

**REFERENCES**

1. Bennett J.E., D., R., Blaser, M.J. Mandell, Douglas, and Bennett's Principles and Practice of Infectious Diseases. (Saunders, Philadelphia, PA; 2014).
